# “Like not having an arm”: a qualitative study of the impact of visitor restrictions on cancer care during the COVID-19 pandemic

**DOI:** 10.1007/s00520-024-08473-8

**Published:** 2024-04-16

**Authors:** Laura M. Holdsworth, Rachel Siden, Bonnie O. Wong, Mae Verano, Anna Sophia Lessios, Holly K. Tabor, Lidia Schapira, Rebecca Aslakson

**Affiliations:** 1 Division of Primary Care and Population Health, Stanford University School of Medicine, Palo Alto, CA USA; 2 Department of Surgery, Brigham and Women’s Hospital, Boston, MA USA; 3 Stanford Cancer Institute, Stanford, CA USA; 4 Department of Anesthesiology, Larner College of Medicine at the University of Vermont, Burlington, VT USA

**Keywords:** COVID-19, Perioperative period, Qualitative methods, Upper gastrointestinal cancer, Decision-making, Caregivers

## Abstract

**Purpose:**

Visitor restriction policies to prevent the spread of COVID-19 among patients and clinicians were widespread during the pandemic, resulting in the exclusion of caregivers at key points of cancer care and treatment decision-making. The aim of this study was to explore how visitor restrictions impacted cancer treatment decision-making and care from patient and physician perspectives.

**Methods:**

Sixty-seven interviews, including 48 cancer patients and 19 cancer and palliative care physicians from four academic cancer centers in the USA between August 2020 and July 2021.

**Results:**

Visitor restrictions that prevented caregivers from participating in clinic appointments and perioperative hospital care created challenges in cancer care that spanned three domains: practical, social, and informational. We identified eight themes that characterized challenges within the three domains across all three groups, and that these challenges had negative emotional and psychological consequences for both groups. Physicians perceived that patients’ negative experiences due to lack of support through the physical presence of caregivers may have worsened patient outcomes.

**Conclusions:**

Our data demonstrate the tripartite structure of the therapeutic relationship in cancer care with caregivers providing critical support in the decision-making and care process to both patients and physicians. Caregiver absences led to practical, psychosocial, and informational burdens on both groups, and likely increased the risk of burnout among physicians. Our findings suggest that the quality of cancer care can be enhanced by engaging caregivers and promoting their physical presence during clinical encounters.

**Supplementary information:**

The online version contains supplementary material available at 10.1007/s00520-024-08473-8.

## Introduction

During the COVID-19 pandemic, visitor restrictions were enacted in many healthcare settings to mitigate the spread of SARS-CoV-2 [1]. While most institutions made exceptions for visitors in certain situations during the pandemic, such as for labor and delivery, or end of life [1], most NCI-designated cancer centers in the USA reported enacting visitor restrictions to both inpatient and outpatient settings, thus barring caregivers from in-person cancer care [2]. While visitor restrictions may reduce the spread of disease [3, 4], previous research from a range of settings has demonstrated that they have numerous negative sequalae, such as reduced satisfaction with care, increased anxiety, depression, and delirium among patients, and increased moral distress among providers [5].

The exclusion of caregivers from cancer care is problematic because family caregivers are integral to cancer patients’ care experience across their cancer journey [6, 7]. The effects of cancer treatments, particularly those for gastrointestinal (GI) cancers, can leave patients with significant symptomology and morbidity [8, 9]. Prior research has identified unmet patient needs in education, access to information, and support services for GI cancers, particularly for underserved populations [10, 11]. Caregivers can help fill these gaps and are an integral part of the patient’s cancer care team, both at home and in the clinical setting, and their presence can enhance cancer care quality [12]. Caregivers support patients in many ways: managing symptoms and side effects of treatment, administering medications, transporting patients to and from appointments, providing emotional support during visits and hospital stays, assisting with physical tasks, and supporting communication and information-sharing with providers, which is crucial for treatment decision-making [6, 13, 14]. While some studies have addressed the impact of visitor restrictions in healthcare broadly [5, 15], less is known about the impact on patients, physicians, and caregivers within cancer care. Therefore, this study aimed to understand how visitor restriction policies during the COVID-19 pandemic impacted cancer care from the perspectives of patients and physicians.

## Methods

### Design

We conducted a qualitative study to explore the impact of the COVID-19 pandemic on cancer care; this analysis focuses on the impact of visitor restrictions on cancer care, including overall experiences and treatment decision-making.

### Participants

Patients were recruited virtually or in-person at an outpatient cancer clinic appointment at one of four academic medical cancer centers across the USA. The cancer centers were located in urban or suburban areas of the US northeast, south, and west (two sites), and often patients traveled from out of the area or nearby states to seek care. All patients were receiving or had recently completed treatment for primary upper GI cancer. This patient population is typically treated surgically and followed by adjuvant chemotherapy and/or radiation. We purposively selected cancer patient participants for maximum variation to gather a range of views reflective of geographic sites, cancer treatment phase, and treatment phase in relation to the pandemic (e.g., surgery pre-pandemic vs during the pandemic) [16]. Participants had to speak either English or Spanish. Physicians from these centers who treat patients with upper GI cancers were also invited to participate in an interview.

### Data collection

Patient interviews were semi-structured and covered the following topics: (1) experiences of coping with cancer care during the pandemic; (2) whether cancer treatments were delayed, deferred, or changed, and how those decisions were made; and (3) use of and preferences regarding telehealth oncologic care (see supplemental material for topic guide). Patient interviews were conducted separately in either English or Spanish by one of four qualitatively trained researchers (LMH, RS, MV, ASL). ASL is a native Spanish speaker and conducted all Spanish language interviews. The topic guide was professionally translated into Spanish and then back translated by ASL to ensure question intention and meaning were aligned. Prior to the interviews, the researchers worked together to align on understanding of the topic guide and interview practices, and interviews were reviewed regularly for quality by LMH who is an experienced qualitative health services researcher.

Physician interviews were also semi-structured and explored the following topics: (1) perceptions of how their patients coped with cancer care during the pandemic; (2) whether/how physicians changed treatments and communicated those decisions to patients; and (3) experiences of using telehealth (see supplemental material for topic guide). Interviews were conducted by one researcher (BOW) with qualitative methods and medical training.

Interviews were conducted via Zoom and were audio recorded with participant permission and professionally transcribed for analysis. Spanish language interviews were transcribed and then translated into English by a professional transcription and translation company; ASL reviewed all translated transcripts while listening to the audio to improve accuracy and indicate areas where verbal cues (e.g., changed intonation) might alter interpretation of the transcript. Interviews continued until no new themes emerged within each group [17].

### Analysis

We took an interpretivist-constructionist approach to analyzing patient and physician experiences of cancer care during the pandemic [18]. Transcripts were uploaded into NVivo (released March 2020) for analysis. Analysis used both deductive and inductive approaches. Initial coding frameworks were developed from the topic guides with a priori codes created to categorize responses to the main question groups (e.g., changes to care, decision-making processes, patient concerns). Three researchers independently coded one transcript each using the initial coding framework, and then performed coding consistency checks and discussed emerging themes not captured in the codebook; this process was repeated twice more. Researchers continued to independently code transcripts and discussed the integration of emergent codes into the codebook as necessary. After interviews were coded, codes were consolidated through discussion and themes were identified. The absence of caregivers during hospital stays and at clinic visits was a recurring emergent theme. Codes related to this experience were further analyzed using a matrix approach to look for patterns in experiences [19]. Separate matrices were created for the patient and physician groups to identify themes specific to each group. Organizing our data in matrices also allowed us to conduct the following quality checks as described by Miles et al. [20]: checking for representativeness, checking for researcher effects, and checking the meaning of outliers.

## Results

Interviews were conducted with 48 cancer patients and 19 cancer care physicians from four academic cancer centers in the USA between August 2020 and July 2021. Three patient interviews were conducted in Spanish. Participant demographics are presented in Table 1. A little more than half (*n* = 27, 56%) of patient interviewees had experienced some or all treatment prior to the start of the pandemic and thus had experience of receiving treatment in the context of no visitor restrictions and in periods of visitor restrictions; for those in survivorship (*n* = 11), these visits were primarily for scans/tests or clinic visits to monitor disease recurrence. Therefore, these patients were able to describe what changes they had experienced to their care due to the pandemic. Physicians included surgical oncologists, medical oncologists, radiation oncologists, and palliative care practitioners.

**Table 1:** Participant characteristics

Characteristic	Patients, *n* = 48	Physician, *n* = 19
Cancer center site (region)		
Site 1 (northeast)	14	2
Site 2 (south)	15	9
Site 3 (west)	17	8
Site 4 (west)	2	0
Gender		
Male	30	13
Female	18	6
Age		n/a
< 50	7	
50–65	19	
65 +	21	
Missing	1	
Race/ethnicity		n/a
White	34	
Hispanic	9	
Asian	2	
Other	3	
Education		n/a
High school or less	11	
College or advanced degrees	27	
Missing	9	
Cancer care received during the pandemic		
Survivorship^1^	11	
Treatment started pre-pandemic, continued during pandemic^2^	16	
Diagnosis and treatment started during pandemic	21	
Specialty	n/a	
Surgical oncologist		8
Medical oncologist		6
Radiation oncologist		3
Palliative care		2
Years in practice	n/a	
0–2 years		3
3 to 9 years		9
10 + years		7

^1^Survivorship = treatment completed, but routine scans/tests and clinic visits for monitoring were ongoing

^2^The start of the pandemic was defined as when local lockdowns/shelter-in-place orders went into effect. The specific date varied by region with all occurring between March 17 and 30, 2020

We identified that participants described their experiences related to the impact of visitor restrictions along two axes: (1) descriptions of care processes that had changed, and (2) emotional or psychological reactions to that change. We present findings related to these two dimensions separately below.

### Care processes

**Fig. 1:**
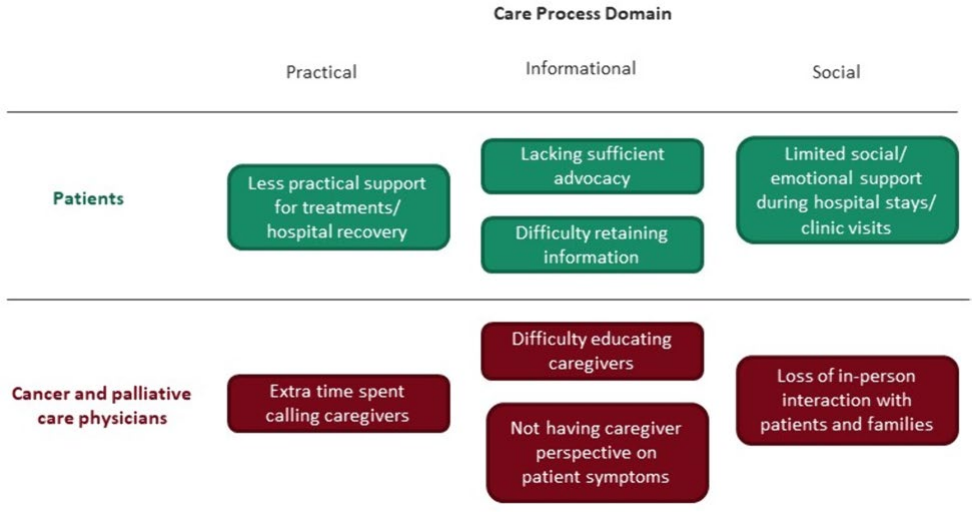
Themes related to the impact of visitor restrictions on care processes for each participant group organized by domain

In relation to changes in care processes, eight themes emerged: four themes for the patient experience and four themes for physicians. Themes identified were organized into three domains: practical, social, and informational aspects of care. Care process themes for each group and their relation to the domain categories are presented in Fig. 1; exemplar quotes for each theme are presented in Table 2.

**Table 2:** Care process thematic domains, themes, and example quotes by participant group

Thematic domain	Patient themes and quotes	Cancer and palliative care physician themes and quotes
Practical	Less practical support during treatments or hospital recovery: “The visitation rules changed when I came out of intensive care. And you know the rules got even more strict and I didn’t see my wife for almost a week. […] You get a little lonely and everything. And if a nurse isn’t there you’re basically doing everything yourself without the help of a loved one. I think it adds a little more stress maybe to you.” (Patient 36)	Extra time spent calling caregivers: “We’ve had to adjust in terms of being more proactive about reaching out to family members, but that does take a lot of time in addition to the patient care that we have to provide.” (Surgical oncologist 6)
Social	Limited social support during hospital stays and clinic visits: “The only thing that was very upsetting to me was I knew my husband couldn’t come be with me because of COVID. I had to go through [surgery] alone. I have to admit that that was really upsetting […] He couldn’t visit at all through my whole recovery. I was in there for seven days. I didn’t have any visitors. I have to tell you it was a very emotional drop off because it was one of those surgeries where you don’t even know if you’re going to come home.” (Patient 15)	Loss of in-person interaction with patients and families: “I still feel like we’re missing some of the really human part of our job, which is the most satisfying part of my job, is meeting the whole family and explaining to them and after surgery, you go out to the waiting room, and you bring them into a conference room and you draw pictures and you’re explaining exactly what you did. […] I can’t really do that on the phone.” (Surgical oncologist 4)
Informational	Lacking sufficient advocacy: “She [daughter] would be a really good advocate. My husband would have been a good advocate too, but she would know what she’s talking about. And, you know, we’re not medical people, but she’s been in that environment for quite a while, and we just felt like it might be the best choice.” (Patient 35)	Difficulty educating caregivers: “[Telehealth is] especially hard for patients who were admitted in the ICU, for example, with their metastatic cancer, multiorgan failure and trying to explain [to family] why they were so sick, but the last time the family saw them, they were doing okay. And so trying to convey how serious things were when they weren’t allowed to see them and they couldn’t see them and couldn’t see the changes in them.” (Palliative care physician 2)
	Difficulty retaining information: “You need to have someone with you when you’re in the hospital and you’re on pain medication. […] I think sometimes you need someone to help you make sense of things, to remember what you want to ask your doctor or nurse. […] You’re really not functioning cognitively the way you should be to make the serious decisions.” (Patient 11)	Not having caregiver perspective on patient symptoms: “Sometimes some patients downplay stuff or they just… I don’t know. Oftentimes it’s like their family or their friends who are there who are asking questions and advocating for them or bringing up to the physician, like the wife will be like, “Okay. He says he’s fine, but he’s actually not.” You know? I worry that some symptoms are missed or side effects aren’t taken seriously.” (Radiation oncologist 3)

#### Patient themes

##### Less practical support during treatments or hospital recovery (*practical domain*)

Patients described having less support for practical aspects of cancer care. Practical support included issues such as having to coordinate transportation to appointments because no one person could stay with the patient during the appointment, not having anyone to bring snacks during long infusions, and having to rely entirely on nurses during hospital stays for personal needs, which may have previously been met by a caregiver.

##### Limited social or emotional support during hospital stays and clinic visits (*social domain*)

Patients described not having social or emotional support during appointments or hospital stays. This was felt acutely in hospitals where patients stayed for days or weeks, and particularly in the context of mask wearing which made some patients feel disconnected from clinicians. Patients described using video calls with families to stay connected with loved ones. In contrast, some patients felt that hospitals and clinics were not suited to socializing and therefore did not have a problem with the absence of visitors.

I definitely miss having somebody with me when I’m sitting, going through chemotherapy. I do miss having [caregiver] there as part of that, as I’m sitting there going through treatment for the four hours or five hours that I’m in the infusion center. The other side of it is, I guess it’s allowed me to concentrate and do work while I’m sitting there too because there’s been no distraction. (Patient 9)

##### Lacking sufficient advocacy (*informational domain*)

Patients who had very involved caregivers described an advocacy role that their caregiver played, which they described as being an additional voice for themselves and someone to help navigate complex cancer care. Patients reflected that their caregiver, when present, would ask many questions and verify that the patient was receiving proper care.

As a patient, when you’re having these discussions about your own health you need some third party there to help you out with it. [...] You don’t have that if you’re there alone and you’re overwhelmed at times by the medical information and what not. (Patient 12)

##### Difficulty retaining information (*informational domain*)

Patients recognized that caregivers were a vital resource for remembering information that they were given during appointments. One patient described his caregiver wife as his “data person” who helped him track his vital signs from all appointments. Caregivers were a second set of eyes and ears and could help the patient process information received during visits. For this reason and during visitor restrictions, patients often voiced preferring telehealth visits over in-person visits so that caregivers could be present and help retain information.

#### Physician themes

##### Extra time spent calling caregivers (*practical domain*)

Physicians described trying to integrate phone calls to update caregivers following surgery or office visits into their daily routine. These calls took up extra time in the physicians’ already busy schedule, and sometimes, it was not feasible.

##### Loss of in-person interaction with patients and families (*social domain*)

Many providers described a diminished joy of practice without caregivers being present. They noted that one of most satisfying aspects of their work was the opportunity to talk to patients and caregivers together in person. This was particularly the case for surgeons who described how their post-operative routines of debriefing and regularly connecting with caregivers had changed, which made it more difficult to communicate and establish relationships.

That feeling like I had to explain myself is not something that I normally have to do, because I normally am so involved with the families during the post-operative care that usually they know the score and are usually at peace with things. And so I will remember those two patients from this time forever […] how it affected their cancer care in the hospital. (Surgical oncologist 5)

##### Difficulty educating caregivers (*informational domain*)

Physicians noted that because caregivers were not physically present to see patients during hospitalization, it was harder to communicate with caregivers about a patient’s condition. Physicians specifically described challenges in communicating about a patient’s decline, or how they were doing following surgery and therefore what their recovery needs would be.

##### Not having the caregiver perspective on patient symptoms (*informational domain*)

Physicians, particularly medical oncologists, described how sometimes patients were reluctant or forgetful in mentioning symptoms, but that caregivers, when present at appointments, could often fill this gap in describing how the patient was managing at home. The lack of caregiver perspective during visitor restrictions was perceived by physicians to negatively impact clinical decision-making.

Many patients will sort of understate their symptoms and say, “No, I’ve been fine”, “I had no nausea”, or “I have no pain”. And all I have to do is look at the family members in the room and watch them roll their eyes, makes me ask more questions. So that’s the body language of the family member and the caregivers [...] As the pandemic went on, they didn’t let family members in rooms. (Medical oncologist 2)

### Emotional and psychological consequences of visitor restrictions

The themes described above encompass challenges in cancer care processes faced by both groups. In describing these challenges, participants also expressed emotional and psychological reactions to these stressors. Patients primarily reported worsened emotional states, such as feeling lonely, bored, and scared during hospital stays or treatments. Patients who had experiences of cancer treatments pre- and post-March 2020 noticed caregiver absences more intensely since they previously had companionship from their caregiver.

You were not allowed to bring anybody with you, so you were alone. You had to be dropped off at the door. You had to wear a mask. You had to be checked in. The whole thing was sort of scary, and that was on the outside just getting in the door. (Patient 7)

Physicians understood that cancer care is difficult for patients, even under the best circumstances, and that caregiver absences magnified these difficulties. They described additional stress in trying to navigate exceptions for visitation policies:

I think the distress level of the patients is higher and the distress level of the providers is higher because no one wants to stand in the way of visitation. The amount of conversations that we have every day, not just palliative care, but oncologists, intensivists, hospitalists around visitation and trying to get exceptions to visitation is a huge part of our days. (Palliative care physician 2)

Physicians noted that patients struggled with isolation, low morale during recovery, and fears and anxiety around dying while in the hospital away from family. Physicians, particularly medical oncologists, described having to cope with the emotional burden of watching patients make difficult decisions alone, whereas normally the patient would have had caregivers with them to help make decisions and provide support.

The number of visitors were severely restricted, and that was extremely hard for our patients too, and for us. I hated it. I understand if a patient’s trying to make a major decision, he or she might want their partner there, and their children there. . . [Patients] rely on their caregivers very intensely and that was a challenging experience. (Medical oncologist 3)

Physicians perceived that the patients’ negative emotional states due to lack of social support may have worsened patient outcomes; one physician of a patient who died in the hospital described how the family felt the patient died of a “broken heart” due to being alone. The stress of providing care in this environment led to physicians expressing fears of burnout.

I think it’s harder to be a doctor during this time because the anxiety level of everybody is so high that the patients are also in need of a lot more support, and it’s not like we have more time or support to give. […] the challenges that have come up because of how difficult giving medical care is just in the world of COVID, I don’t think is actually addressed much. And I think that it’s really ripe for burnout. (Medical oncologist 1)

## Discussion

Our analysis identified that COVID-19-induced visitor restrictions to hospitals and clinics negatively impacted practical, informational, and social aspects of cancer care for both patients and physicians. Our findings echo those of a previous study which found that the absence of visitors following surgery negatively affected psychosocial well-being [21], but in addition, our study found more negative effects related to practical and informational aspects of care. Of note, the absence of caregivers had multiple psychosocial effects on physicians who felt reduced connection with patient’s families and perceived their patients had a harder time coping with treatment. This is perhaps not surprising as physicians had extensive experience of cancer care prior to the pandemic and may have felt the difference more acutely as compared to patients who likely had limited prior experience of cancer care and thus were not aware of what may have been missing. The distinct impact of the lack of caregiver presence felt by physicians demonstrates the unique role that caregivers have in the therapeutic relationship in cancer care between physicians and patients.

Though visitor restrictions were recognized as an important risk mitigation strategy for COVID-19 during the pandemic, many physicians in our study expressed distress at their implementation. The stressors from visitor restrictions occurred within a context of healthcare that was already under immense pressure from pandemic impacts, such as high patient volumes during surges, personal protective equipment shortages, changing guidelines for care and treatment, and fear of virus transmission [22]. Our findings indicate that visitor restrictions added to physician burden due to additional time spent connecting with and educating caregivers, making clinical decisions without input from caregivers, and perceiving patient distress due to lack of social and emotional support. Prolonged periods of exposure to moral stressors, such as caring for patients without family contact or accompanying patients dying alone, can lead to moral distress or injury [23]. Our findings add nuance to the literature about high levels of burnout and distress experienced by physicians during the pandemic [24]. The negative impact of visitor restrictions is concerning as oncologists and palliative care providers may already be at increased risk of “compromised well-being due to the nature and intensity of the clinical stresses they face” [25], pp. 2. Additionally, such restrictions do not account for the fact that patients and caregivers frequently travel and cohabitate, and thus may have done unnecessary harm [7].

Many patients in our study, particularly those in survivorship, reported having visits with physicians via telehealth. There is abundant literature about the perceived value and role of touch in creating a human connection between patients and their providers [26–29]. Touch is a non-verbal form of communication that is particularly relevant in serious illness care for expressing empathy during distressing encounters and trust building. During the pandemic, the reliance on telehealth modalities of care and increased social distancing in general precluded the use of touch in many instances. However, while physicians perceived that this contributed to feeling unable to connect with patients, patients did not generally mirror this sentiment. Rather, patients expressed that physical distance with their caregivers during visitor restrictions during hospital stays or for clinic visits was emotionally distressing. This suggests that ensuring patients can be physically present with their caregivers during clinic visits and hospital stays may be more protective for mental and emotional well-being than being physically present with their cancer care practitioner. Evidence from research with older adults indicates that the participation of caregivers during clinical visits can enhance patient satisfaction with care, including perception of provider technical skill and quality of communication [30]. Another study found that 60% of oncology patient users of an online health portal preferred joint communication with their caregiver and provider [31]. Our findings, along with prior research [12], suggest that the quality of cancer care can be enhanced by engaging caregivers and promoting their physical presence during clinical encounters, regardless of modality.

This study had several strengths and limitations. A strength of this study was recruiting participants from multiple sites across different regions and institutions; themes were common across sites indicating that they are likely transferable to similar settings. We noted a divergence in the depth of experiences between patients who had started treatment prior to the pandemic, and those who started during the pandemic; those who started prior were able to describe direct, pandemic-related care changes. Those who started treatment during the pandemic and with no prior cancer experience were unable to compare pre- and post-pandemic experiences, and instead described what they did not like about their care. Only one researcher (ASL) spoke Spanish fluently and therefore the other researchers were not able to utilize the audio to familiarize themselves with the data prior to coding; this may have led to certain nuance being missed during coding. Our practitioner group included only physicians, and therefore our findings are likely limited to that professional group.

Our study indicates that visitor restrictions were detrimental to not just patients and caregivers, but also cancer care physicians. Caregivers fill a clearly independent role in the care process and their absence particularly impacted physicians’ joy of practice during the pandemic. Whether under pandemic conditions or not, health systems should consider how well they engage caregivers across different clinical encounter modalities as they are critical to the patient and physician experience of quality cancer care [12]. Team-based approaches to care in which the diversity of the caregiver perspective is recognized and sought out as a valuable contribution to interprofessional care may be one approach to elevate the caregiver voice and enhance cancer care quality for all involved [32]. However, little research has been done to date on how to formally integrate caregivers into team-based approaches to care [33]. Findings from our study and prior research indicate that future work on implementing team-based approaches should emphasize information exchange, particularly as it influences clinical decision-making and patient satisfaction [10, 12].

### Supplementary information

Below is the link to the electronic supplementary material.

Supplementary file1 (DOCX 33 KB)

## Data Availability

No datasets were generated or analyzed during the current study.
