# Supplementary material for: “Like not having an arm”: a qualitative study of the impact of visitor restrictions on cancer care during the COVID-19 pandemic
Source: Support Care Cancer. 2024 Apr 16;32(5):288. doi: 10.1007/s00520-024-08473-8 (PMC11018646; doi:10.1007/s00520-024-08473-8)
Supplement: Supplementary file 1 — Supplementary file1 (DOCX 33 KB) [file 520_2024_8473_MOESM1_ESM.docx]

**Patient Interview Guide**

| **Main Topic** | **Prompts/probes** |
| --- | --- |
| I first want to start with some background information about your cancer care. | |
| - When were you diagnosed with cancer? - When did you have surgery? - What other treatment have you had? |  |
| Thank you for that background information. I now want to ask about what your care has been like since the start of the pandemic in March 2020. | |
| *Skip if all care has been during the pandemic*  I want you to try to remember the first visit or appointment you had for your cancer care that took place after the start of the pandemic when lock down first started and people were taking precautions for the virus. Can you tell me what that first appointment was like? | What was the appointment for?  What went well?  What could have been better?  *If they had care before and after pandemic*: How was this visit different to previous visits before the pandemic? |
| Overall, how well do you feel your cancer care has gone during the pandemic? | Why do you feel that way? |
| Have any of your cancer treatments changed due to the pandemic? | How did the treatments change?  How were the decisions made to change the cancer treatment decisions?  How did you feel about the changes that were made?  Were you given any options or choices for your care? |
| Did you have any visits by video or telephone?  (I*f yes):* What did you like about video/telephone/virtual visits? | Is there anything you didn’t like about virtual visits?  Did you have any virtual visits for your cancer care before the pandemic?  In future, when would you prefer to have a virtual or telephone visit, versus seeing the doctor in person? Why? |
| How well has the treatment you’ve received met your expectations? | Is there anything that could be done to help make getting cancer treatment easier?  Do you think the pandemic has affected the likely success of your treatment?  *If they had to come to the hospital/ infusion center/doctor’s office*: Did you feel safe coming to the [hospital/ infusion center/ doctor’s office] during the pandemic? What made you feel safe/ not safe? |
| As a patient undergoing cancer care, how well do you feel that you’ve been able to cope with your care needs during the pandemic? | What are your strategies for coping?  *If they had cancer before the pandemic*: Has the way you cope with your cancer care needs changed since the start of the pandemic? How/in what ways?  Is there anything that your care team could have done to better meet your needs? |
| Knowing what you know now about what it’s like to be going through cancer treatment/survivorship during a pandemic, what would you say could have been improved? | Is there anything your cancer team could have done differently? |
| Has your doctor talked to you about getting the COVID-19 vaccine? | *If* yes: How did your doctor’s opinion of the vaccine affect your choice to get it /not get it?  Have you changed any precautions you take after receiving the vaccine? What has made you feel able/ not able to change your precautions? |
| Is there anything else about your cancer care that I haven’t asked about that you think I should know? | |

**Practitioner Interview Guide**

| **Main Topic** | **Prompts/probes** |
| --- | --- |
| What is a general week of practice like? | What patients do you see?  How are patients typically referred to you (e.g. medical oncologist, other surgeons, etc.)? |
| **I now want to talk about what it has been like providing cancer care during the COVID-19 pandemic.** | |
| How has the COVID-19 pandemic changed how you provide cancer care? | Were there delays or changes in cancer care? If so, how were the decisions for delays or changes made? How were these decisions communicated to cancer patients? |
| What has changed during the pandemic about how you interact with or communicate with patients? | Are you using telehealth visits? How has that been?  Are you communicating more or less with your patients? |
| What have been your patients’ concerns regarding cancer care during the COVID-19 pandemic? |  |
| What have been your concerns regarding cancer care during the COVID-19 pandemic? | What have been some of your emotions or feelings around providing cancer care during the COVID-19 pandemic? |
| How have decisions regarding cancer treatment been made during this epidemic? | How should decisions regarding cancer treatment be made during a pandemic? |
| Is there anything else you’d like to tell me about the experience of delivering cancer care during the COVID-19 Pandemic? | |
